# Evaluation of effects of autologous fat grafting on aesthetic outcomes in breast reconstruction following mastectomy: Effects of reconstruction type and prior radiotherapy exposure

**DOI:** 10.1016/j.jpra.2026.05.027

**Published:** 2026-05-19

**Authors:** Sarah Omar, Abdou M.A. Darwish, Krzysztof Sosnowski, Ayman El-Henawy, Ashraf Othman, Charles Malata

**Affiliations:** a Department of Plastic Surgery, Minia University, Egypt; b School of Clinical Medicine, University of Cambridge, Cambridge, United Kingdom; c Plastic surgery Department, Cairo University, Egypt; d Clinical Pathology department, Minia University, Egypt; e Department of Plastic and Reconstructive Surgery, Hospital, Cambridge University Hospitals NHS Foundation Trust, Cambridge, United Kingdom; f Anglia Ruskin University School of Medicine, Cambridge & Chelmsford, United Kingdom; g Cambridge Breast Unit, Addenbrooke’s Hospital, Cambridge, United Kingdom

**Keywords:** Autologous fat grafting, Breast reconstruction, Radiotherapy, BCCT.core, Harvard scale, Implant-based reconstruction

## Abstract

**Background:**

Autologous fat grafting improves breast reconstruction aesthetics, yet impact relative to natural post-reconstructive changes remains poorly characterized.

**Methods:**

Retrospective analysis of 92 reconstructions (42 fat-grafted, 50 non-grafted) using BCCT.core and Harvard Scale assessments at 6–12 months.

**Results:**

Non-grafted reconstructions demonstrated significant deterioration (46%; BCCT.core *p* = 0.027, Harvard *p* < 0.001) uniformly across modalities. Conventional 3-week radiotherapy produced greater deterioration than 5-day regimens (*p* = 0.036, *p* = 0.024). Fat grafting reversed this trajectory, achieving 88.1% versus 4% complete complaint resolution (Mann-Whitney *p* < 0.001; effect sizes 0.41–0.84) across all reconstruction types and radiotherapy exposures. Irradiated reconstructions demonstrated greatest gains. One to two sessions typically sufficed.

**Conclusion:**

Post-reconstructive deterioration should be anticipated. Fat grafting functions as regenerative intervention modifying biological maturation. Findings support proactive staged incorporation into algorithms, particularly for irradiated patients.

## Introduction

Autologous fat grafting has become an established adjunct in breast reconstruction, providing volumetric augmentation and biologically mediated tissue quality improvement.^1^, ^2^, ^3^ In post-mastectomy reconstruction, particularly following radiotherapy (PMRT), fat grafting improves texture, pliability, and vascularity of irradiated tissues while facilitating contour optimization.^4^, ^5^, ^6^ Regenerative effects attributed to adipose-derived stem cells reduce radiation-induced fibrosis and enhance tissue pliability and neovascularization, while avoiding immunogenicity and foreign material risks.^2^^,^^7^

Despite widespread use, aesthetic benefits have not been consistently quantified using validated objective measures.^6^^,^^9^^,^^10^ Critically, existing literature reports improvement in fat-grafted reconstructions without comparative assessment of natural aesthetic trajectory in non-grafted reconstructions^15^, ^16^, ^17^ This absence of control cohorts limits understanding of whether observed benefits represent active improvement, prevention of expected deterioration, or both. Furthermore, differential impact of radiotherapy fractionation regimens on aesthetic maturation in grafted versus non-grafted reconstructions remains poorly characterized.^12^^,^^14^ Clarification of natural post-reconstructive aesthetic trajectory and its modification by fat grafting is essential for evidence-based counseling, timing of interventions, and interpretation of outcome studies.

This study evaluated autologous fat grafting impact on aesthetic outcomes following breast reconstruction, with specific assessment of contour, symmetry, and overall cosmetic appearance. Additionally, the influence of reconstruction modality and prior radiotherapy exposure on aesthetic outcome was examined.

## Methods

This retrospective cohort study analyzed 92 breast reconstructions in 2 cohorts from Addenbrooke's Hospital, Cambridge (July 2024–July 2025), using EPIC electronic health records.^8^ All patients underwent mastectomy and breast reconstruction for breast cancer. Cohort 1 (*n* = 42) received ≥ 1 autologous fat grafting session (September 2022–December 2024). Cohort 2 (*n* = 50) received no fat grafting. Patients in both cohorts were selected based on patients’ complaint from shape deformity or volume/shape asymmetry which was confirmed by examination at the time.

### Fat grafting technique (Cohort 1)

Patients were marked preoperatively. Donor sites (lower abdomen, flanks, thighs) were infiltrated with tumescent solution at 1:1 wet-to-superwet ratio with vasoconstriction latency. Adipose tissue was harvested via low-negative-pressure liposuction (≤−0.5 atm) using subcutaneous fanning technique. Harvested fat underwent centrifugation (3000 rpm, 3 min). Fat was injected via stab incisions using blunt cannulas with multilayered retrograde technique, small aliquots, and low injection pressure, targeting marked deficiencies. Injection volumes ranged 80–300 mL per breast. Incisions were closed with sutures without drains. Donor sites received compression garments.

### Evaluation of aesthetic outcome

Aesthetic outcomes were evaluated using: (1) primary complaint resolution, (2) objective BCCT.core assessment, and (3) subjective Harvard scale assessment.^9^^,^^10^ Assessments used photographs at 6–12 months after final fat grafting (Cohort 1) or reconstructive procedure (Cohort 2), allowing tissue stabilization and scar maturation.

Complaint resolution was assessed from patient feedback. Cohort 1 complaints were those prompting fat grafting; Cohort 2 complaints regarding breast shape, volume, or symmetry were documented in records. Resolution was categorized as complete, partial, or none.

BCCT.core software assessed asymmetry, skin color/texture, and scar visibility, generating grades: poor, fair, good, excellent.^9^ Standardized frontal photographs were analyzed for comparisons (fat grafting status, radiotherapy exposure, reconstruction type). 'Fat Grafting Effect Score (BCCT.core)' for Cohort 1 and 'Maturation Effect Score (BCCT.core)' for Cohort 2 quantified grade changes as positive/negative integers.

Using Harvard scale, 3 plastic surgeons independently evaluated frontal/oblique photographs at pre-first fat grafting and 6–12 months post-final session (Cohort 1); 1 month and 6–12 months post-final procedure (Cohort 2). Grading (excellent, good, fair, poor) was relative to contralateral breast. Consensus required ≥ 2 evaluators; scores were otherwise averaged. 'Fat Grafting Effect Score (Harvard)' for Cohort 1 and 'Maturation Effect Score (Harvard)' for Cohort 2 represented net rating changes as positive/negative integers.

Statistical analyses used JASP software.^11^ Between-group comparisons employed Kruskal-Wallis tests for non-parametric data. Categorical associations were examined via ordinal/nominal regression. Significance threshold: *P* < 0.05.

## Results

### Patient profiles

Patient demographics and clinical characteristics are presented in Table 1.

**Table 1:** Patient demographics and clinical characteristics for fat-grafted (Cohort 1) and non-fat-grafted (Cohort 2) breast reconstructions.

Characteristic	Cohort 1 (*n* = 42) Fat-Grafted Reconstructions	Cohort 2 (*n* = 50) Non-Fat-Grafted Reconstructions
Patients	32	48
Bilateral Reconstructions	10 (23.8%)	2 (4%)
Age (years)		
Range	36–63	24–70
Mean (Median)	48.9 (49)	52.5 (53)
Mastectomy Type		
Skin-sparing	31 (73.8%)	49 (98%)
Nipple-sparing	5 (11.9%)	0
Skin-reducing	4 (9.5%)	1 (2%)
Simple	2 (4.8%)	0
Reconstruction Sequence		
Primary	30 (71.4%)	49 (98%)
Secondary	6 (14.3%)	1 (2%)
Tertiary	6 (14.3%)	0
Reconstruction type		
DIEP	16 (38.1%)	20 (40%)
Implant-based	14 (33.3%)	15 (30%)
LD	12 (28.6%)	15 (30%)
Prior radiotherapy Exposure	17 (40.5%)	26 (52%)
3-week fractionation	14 (82.3%)ᵃ	17 (34%)
5-day ultrahypofractionated	3 (17.6%)ᵃ	9 (18%)
Fat grafting indications		
Contour deformities	26 (61.9%)	
Volume asymmetry	14 (33.3%)	
Thin mastectomy flaps	7 (16.6%)	
Rippling	5 (11.9%)	
Tissue fibrosis/tightness	4 (9.5%)	
Other	4 (9.5%)ᵇ	
Number of Grafting sessions		
One	22 (52.3%)	
Two	13 (30.9%)	
Three	7 (16.6%)	
Harvest sites		
Anterior abdominal wall	25 (59.5%)	
Flanks	16 (38.1%)	
Thighs	6 (14.2%)	
Other	6 (14.3%)ᶜ	

DIEP, deep inferior epigastric perforator; LD, latissimus dorsi.

ᵃ Percentage of those receiving radiotherapy within cohort.

ᵇ Includes contracted lower pole (*n* = 2), breast sagging (*n* = 1), accentuated anterior axillary fold crease (*n* = 1).

ᶜ Includes outer buttocks/mons pubis (*n* = 4), back rolls (*n* = 1), anterior axillary bulge (*n* = 1).

### Interobserver reliability of the Harvard scale

Interobserver reliability of the Harvard Scale demonstrated good agreement (weighted Cohen's κ = 0.61, *p* < 0.001), supporting the overall consistency of the subjective assessments.

### Complaint resolution

In cohort 1, 37 of 42 reconstructions (88.1%) achieved complete complaint resolution, 5 (11.9%) partial improvement; no cases showed lack of response or worsening. Conversely, cohort 2 showed markedly different outcomes: 23 of 50 (46%) worsened, 20 (40%) showed no change, and 7 (14%) improved, 5 (10%) partial resolution, 2 (4%) complete resolution.

### Aesthetic evaluation of both cohorts

#### Cohort 1

**Fig. 1:**
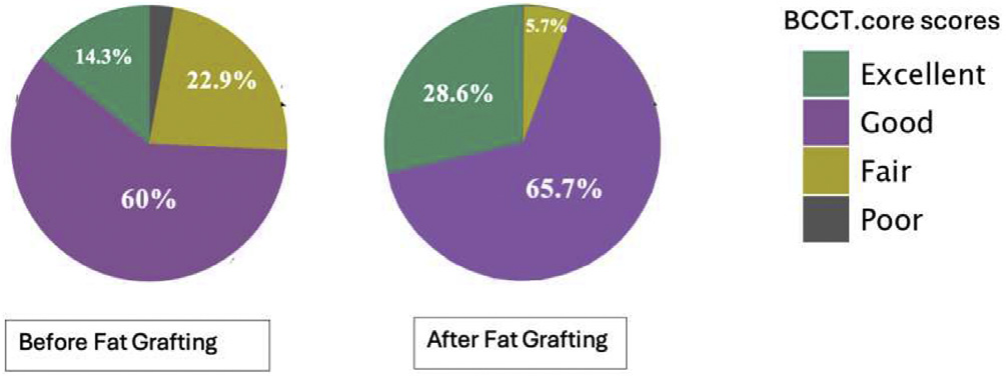
Pie charts displaying distribution of frequencies of BCCT.core scores among reconstructions before and after fat grafting (Cohort 1).

**Fig. 2:**
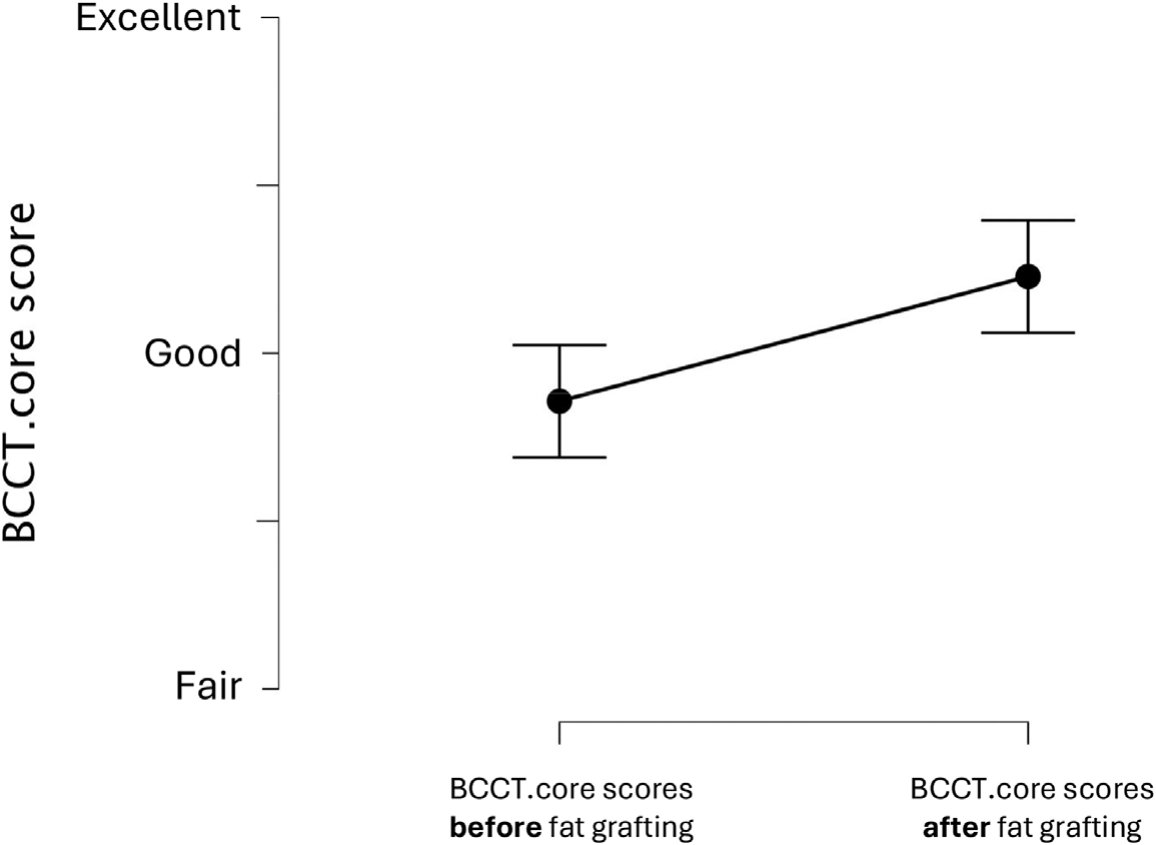
A paired line plot showing an upward trajectory, indicating improved BCCT.core scores after fat grafting among reconstructions in Cohort 1. The Wilcoxon Signed-Rank Test demonstrated that this improvement was statistically significant (W = 5.000, Z = 2.667, *p* = 0.006).

Cohort 1 BCCT.core assessment (35/42 reconstructions). Pre-grafting: Good (*n* = 21, 60%), Fair (*n* = 8, 22.8%), Excellent (*n* = 5, 14.2%), Poor (*n* = 1, 2.8%); mode=Good. Post-grafting: Good (*n* = 23, 65.7%), Excellent (*n* = 10, 28.6%), Fair (*n* = 2, 5.7%); no Poor (Fig. 1); mode=Good. BCCT.core Fat Grafting Effect Score: no change (*n* = 23, 65.7%), one-grade improvement (*n* = 8, 22.8%), two-grade improvement (*n* = 3, 8.5%), one-grade deterioration (*n* = 1, 2.8%). Wilcoxon Signed-Rank Test demonstrated significant improvement (W = 5.000, Z=−2.667, *p* = 0.006), with mean scores increasing from 2.86±0.69 to 3.23±0.55 and strong effect size (rank-biserial correlation=−0.872). (Fig. 2).

**Fig. 3:**
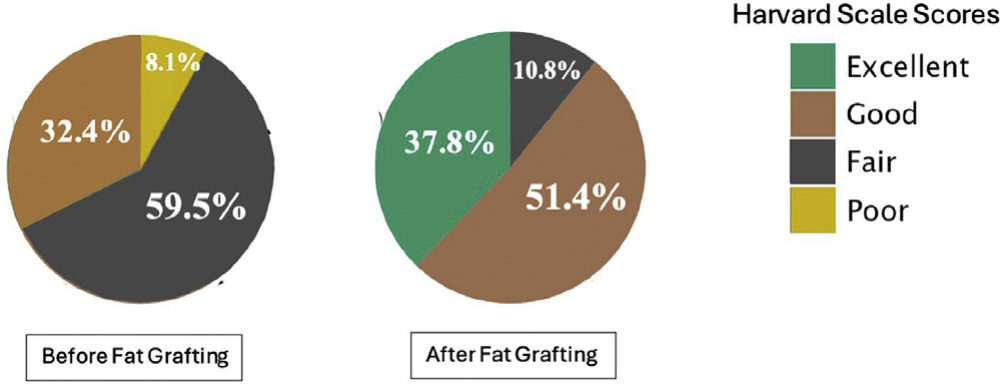
Pie charts displaying distribution of Harvard scale scores of reconstructions in Cohort 1 before and after fat grafting.

Harvard scale evaluation (37/42 reconstructions). Pre-grafting: Fair (*n* = 22, 59.5%), Good (*n* = 12, 32.4%), Poor (*n* = 3, 8.1%); mode=Fair. Post-grafting: Good (*n* = 19, 51.4%), Excellent (*n* = 14, 37.8%), Fair (*n* = 4, 10.8%); mode=Good. (Fig. 3).

**Fig. 4:**
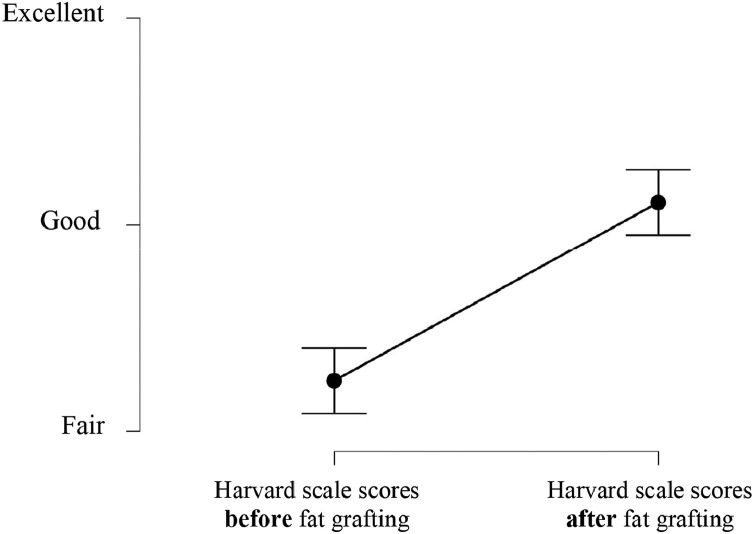
A paired line plot showing an upward trajectory, indicating significantly improved Harvard scale aesthetic outcomes of reconstructions in Cohort 1 after fat grafting. The Wilcoxon Signed-Rank Test demonstrated that this improvement was statistically significant (*p* < 0.001).

Harvard Fat Grafting Effect Score: no change (*n* = 5, 13.5%, score=0), one-grade improvement (*n* = 26, 70.3%, score=+1), two-grade improvement (*n* = 6, 16.2%, score=+2). Wilcoxon Signed-Rank Test demonstrated significant improvement (W = 0.000, Z = 4.457, *p* < 0.001), with means rising from 2.24±0.60 to 3.11±0.81. Rank-biserial correlation=−1.000 denotes large effect size, indicating statistically and clinically robust improvement. (Fig. 4).

#### Cohort 2

**Fig. 5:**
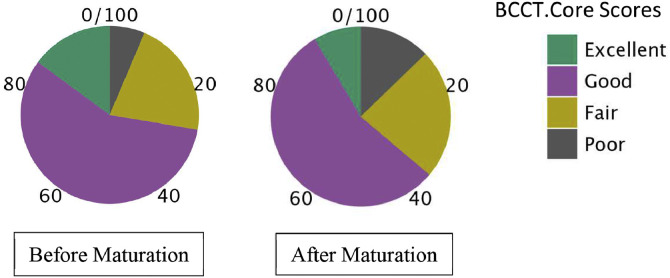
Pie charts displaying distribution of BCCT.Core scores among non-fat-grafted reconstructions (Cohort 2) before and after scar maturation and oedema resolution.

Cohort 2 BCCT.core assessment (47/50 reconstructions). One month post-reconstruction: Good (*n* = 27, 57.4%), Fair (*n* = 10, 21.3%), Excellent (*n* = 7, 14.9%), Poor (*n* = 3, 6.4%). At 6–12 months: Good (*n* = 26, 55.3%), Fair (*n* = 11, 23.4%), Poor (*n* = 6, 12.8%), Excellent (*n* = 4, 8.5%). (Fig. 5)

**Fig. 6:**
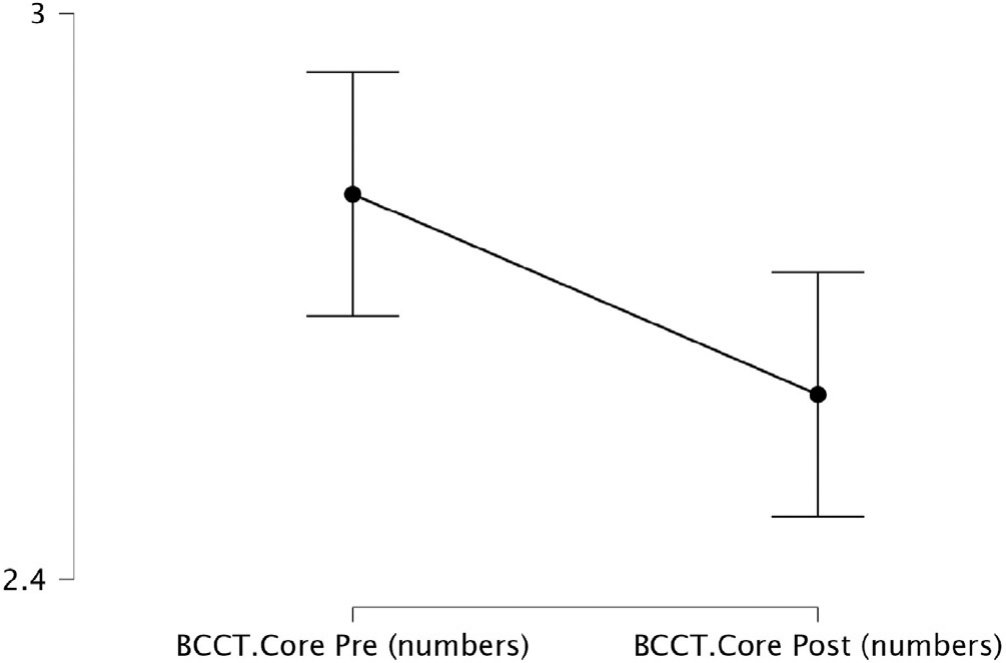
A paired line plot showing deterioration of aesthetic outcomes indicated by lower BCCT.Core scale scores of reconstructions in Cohort 2 scar maturation. The Wilcoxon Signed-Rank Test demonstrated that this deterioration was statistically significant (*p* = 0.027).

BCCT.core Maturation Effect Score: no change (*n* = 27, 57.4%, score=0), one-grade deterioration (*n* = 15, 31.9%, score=−1), one-grade improvement (*n* = 5, 10.6%, score=1). Wilcoxon Signed-Rank Test demonstrated significant deterioration (W = 157.5, Z = 1.96, *p* = 0.027), with means falling from 2.80±0.77 to 2.59±0.82 and large effect size (rank-biserial correlation=0.5). (Fig. 6).

**Fig. 7:**
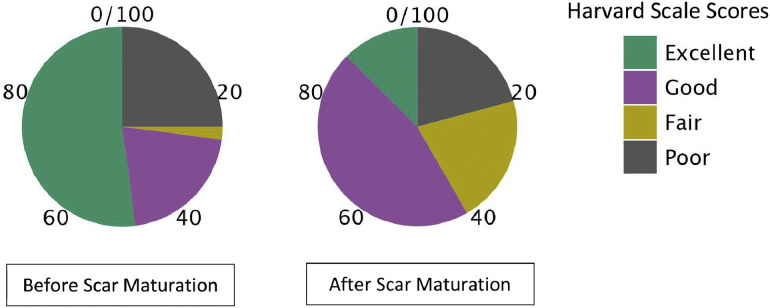
Pie charts displaying distribution of Harvard scale scores of reconstructions in Cohort 2 before and after scar maturation and oedema resolution.

Harvard scale evaluation (48/50 reconstructions). One-month post-reconstruction: Good (*n* = 25, 52.1%), Excellent (*n* = 12, 25%), Poor (*n* = 1, 2.1%); mode=Good. At 6–12 months: Good (*n* = 22, 45.8%), Fair (*n* = 10, 20.8%), Poor (*n* = 10, 20.8%), Excellent (*n* = 6, 12.5%); mode=Good despite shift. (Fig. 7)

Harvard Scores Pre (numbers) - Harvard Scores Post (numbers)

**Fig. 8:**
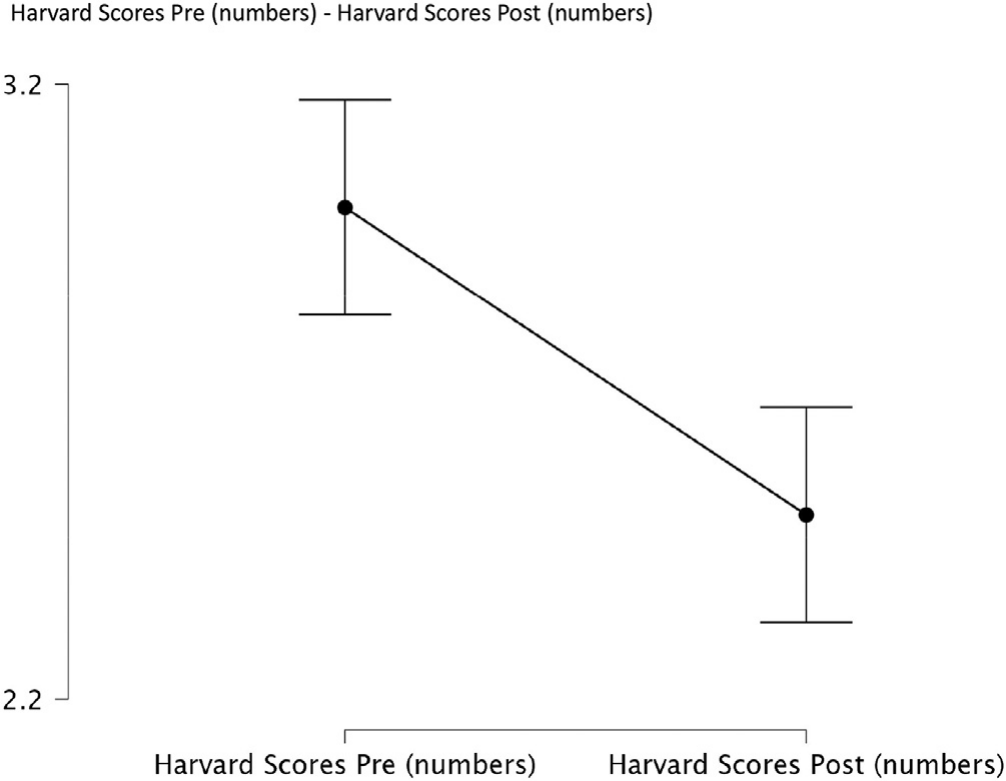
Deterioration in aesthetic outcomes during scar maturation in Cohort 2. Paired analysis demonstrates statistically significant decline in Harvard Scale scores between 1 month and 6–12 months post-reconstruction (Wilcoxon Signed-Rank Test, *p* < 0.001). Each line represents one reconstruction.

Harvard Maturation Effect Score: one-grade deterioration (*n* = 20, 41.7%, score=−1), no change (*n* = 19, 39.6%, score=0), one-grade improvement (*n* = 5, 10.4%, score=+1), two-grade deterioration (*n* = 3, 6.3%, score=−2), three-grade deterioration (*n* = 1, 2.1%, score=−3). Wilcoxon Signed-Rank Test demonstrated significant deterioration (W = 370, Z = 3.29, *p* < 0.001), with means falling from 3 ± 0.74 to 2.5 ± 0.96. Rank-biserial correlation=0.7 denotes large effect size, indicating statistically and clinically robust deterioration. (Fig. 8)

### Comparison between effect scores of both cohorts

**Fig. 9:**
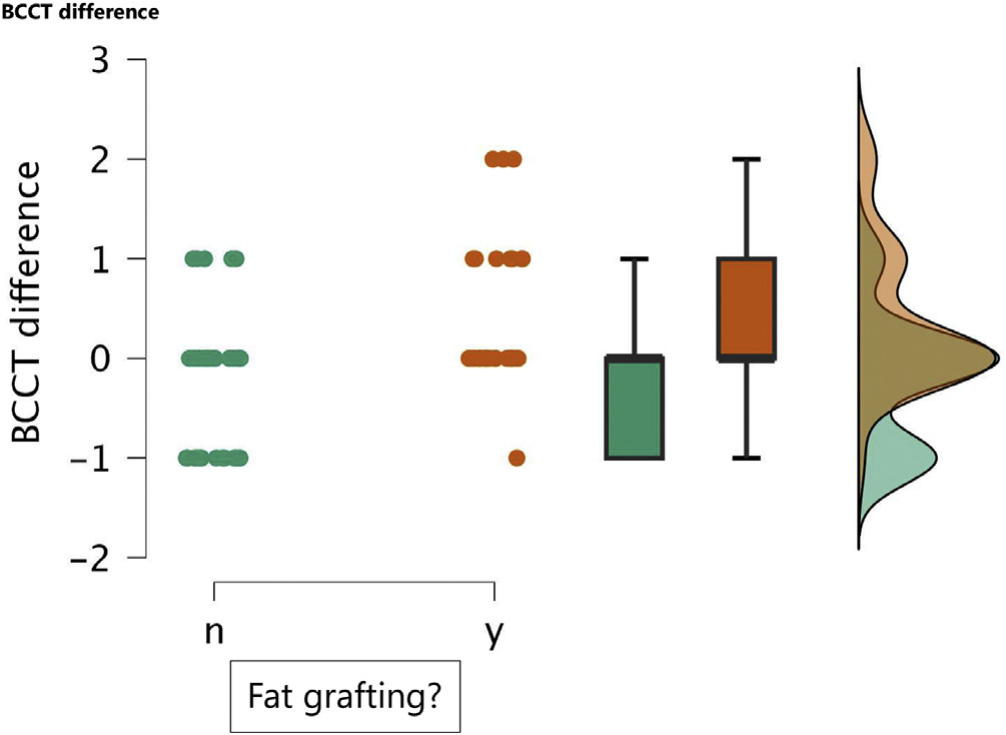
Raincloud plot illustrating the differential outcomes between fat-grafted and non-fat-grafted reconstructions using BCCT.core assessment. Fat grafting effect scores (Cohort 1) are compared with maturation effect scores (Cohort 2), demonstrating significant aesthetic improvement attributable to lipofilling versus progressive deterioration in untreated reconstructions. Statistical comparison using Mann-Whitney U test confirmed a highly significant difference between cohorts (*p* < 0.001).

Mann-Whitney testing comparing Cohort 1′s BCCT.core Fat Grafting Effect Score with Cohort 2′s BCCT.core Maturation Effect Score demonstrated significant difference (*p* < 0.001) with medium effect size (0.41). Mean ranks (fat grafting 51.14 vs. no-fat grafting 34.32) indicated greater improvement with fat grafting (Fig. 9).

**Fig. 10:**
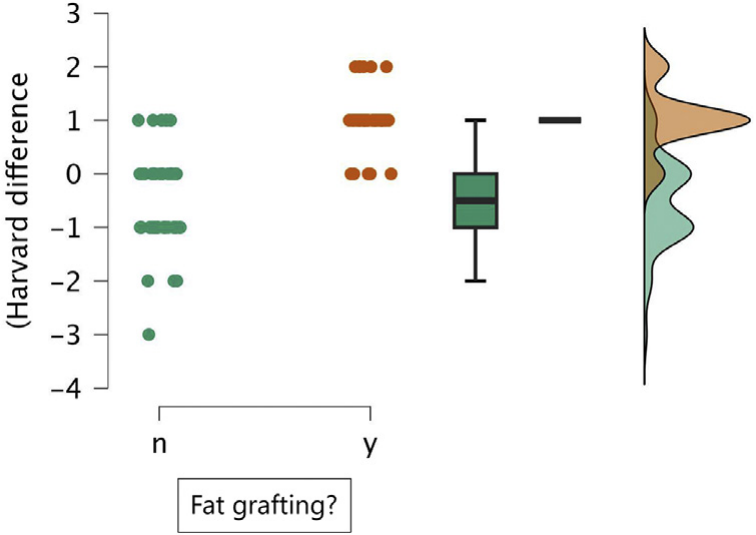
Raincloud plot comparing Harvard Scale-derived fat grafting effect scores (Cohort 1) with maturation effect scores (Cohort 2). The fat grafting effect scores demonstrate aesthetic improvement following lipofilling, in contrast to the deterioration observed in non-fat-grafted reconstructions during scar maturation. Mann-Whitney U test revealed a statistically significant difference between groups (*p* < 0.001).

Mann-Whitney testing comparing Harvard Fat Grafting Effect Score with Harvard Maturation Effect Score demonstrated significant difference (*p* < 0.001) with larger effect size (0.84). Mean ranks (fat grafting 63.28 vs. no-fat grafting 27.36) indicated greater subjective improvement with fat grafting (Fig. 10).

### Effect of reconstruction modality on aesthetic outcome achieved in each cohort

Effect of reconstruction modality on aesthetic outcome was examined using BCCT.core and Harvard-derived Fat Grafting Effect Scores for Cohort 1, and corresponding Maturation Effect Scores in Cohort 2.

Cohort 1 BCCT.core: DIEP showed 50% no change, 37.5% one-grade improvement; implant-based showed 57.1% unchanged, 14.3% two-grade improvement, 7.1% one-grade decline; LD showed 50% no change, 16.7% one-grade improvement, 8.3% two-grade improvement. Harvard scale: DIEP showed 93.8% one-grade improvement, 6.3% unchanged; implant-based showed 42.9% one-grade improvement, 28.6% two-grade improvement, 14.3% no change (14.3% missing); LD showed 41.7% one-grade improvement, 16.7% two-grade improvement, 16.7% unchanged (25% missing). Kruskal-Wallis testing demonstrated no significant relationship between reconstruction type and BCCT.core Fat Grafting Effect Score (*p* = 0.847) or Harvard Fat Grafting Effect Score (*p* = 0.559).

Cohort 2 BCCT.core: DIEP showed 84.2% no change, 10.5% one-grade deterioration, 5.3% one-grade improvement; implant-based showed 46.7% one-grade deterioration, 33.3% unchanged, 20% one-grade improvement; LD showed 46.2% no change, 46.2% one-grade deterioration, 7.7% one-grade improvement (missing: 5% DIEP, 13.3% LD). Harvard scale: DIEP showed 68.4% no change, 26.3% one-grade deterioration, 5.3% one-grade improvement; implant-based showed 46.7% one-grade deterioration, 20% one-grade improvement, 13.3% no change, 13.3% two-grade deterioration, 6.7% three-grade deterioration; LD showed 57.1% one-grade deterioration, 28.6% no change, 7.1% one-grade improvement, 7.1% two-grade deterioration (missing: 5% DIEP, 6.7% LD). Kruskal-Wallis testing demonstrated no significant relationship between reconstruction type and BCCT.core Maturation Effect Score (*p* = 0.315) or Harvard Maturation Effect Score (*p* = 0.156).

### Fat grafting effect scores, maturation effect scores and radiotherapy

**Fig. 11:**
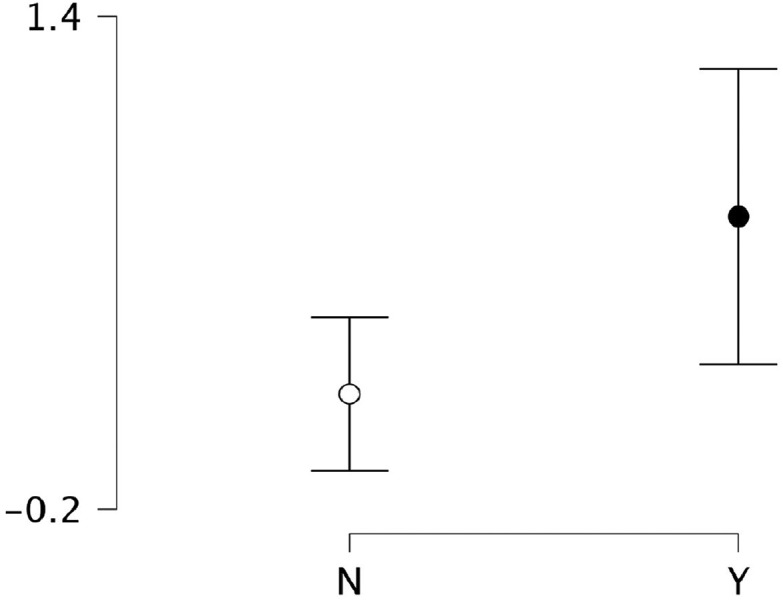
Paired line plot illustrating BCCT.core-derived fat grafting effect scores in Cohort 1, stratified by radiotherapy status. A statistically significant difference was observed between patients who received radiotherapy and those who did not (Mann-Whitney U test, *p* = 0.015).

In Cohort 1, Mann-Whitney U testing assessed radiotherapy influence on fat grafting efficacy. Harvard scale scores showed no significant difference between irradiated and non-irradiated reconstructions (U = 167.000, *p* = 0.830; mean ranks 18.57 vs. 19.26). BCCT.core-derived fat grafting effect scores differed significantly (U = 78.500, *p* = 0.015), with higher mean ranks in irradiated reconstructions (22.96 vs. 15.41), suggesting greater objective improvement in RT-exposed groups (Fig. 11).

BCCT.core Fat Grafting Effect Scores varied by radiotherapy group. In 3-week RT cohort (*n* = 14), 28.6% improved one grade, 28.6% two grades, 28.6% unchanged. In 5-day RT group (*n* = 3), 33.3% improved one grade, with no two-grade improvement or deterioration. Among non-irradiated reconstructions (*n* = 25), 72% unchanged, 12% improved one grade, 4% two grades, 4% declined one grade. Harvard scale showed improvement across all groups: 3-week RT demonstrated 57.1% one-grade, 14.3% two-grade improvement, 14.3% unchanged; 5-day RT showed 66.7% one-grade improvement with no two-grade changes or deterioration; non-irradiated reconstructions showed 64% one-grade, 16% two-grade improvement, 12% unchanged.

RT exposure was not associated with fat grafting session number (Kruskal-Wallis, *p* = 0.843; stratified *p* = 0.197). Ordinal regression using BCCT.core-based scores demonstrated significant RT dose effect on post-grafting improvement (χ²=6.405, *p* = 0.041); absence of RT was associated with significantly less improvement versus 3-week RT (*p* = 0.024). No significant RT dose effect was observed using Harvard scale.

In Cohort 2, Mann-Whitney U testing demonstrated no significant RT effect on BCCT.core-derived Maturation Effect Scores (U = 329.000, *p* = 0.205; mean ranks 26.3 vs. 21.7) or Harvard-derived scores (U = 347.000, *p* = 0.187; mean ranks 27.11 vs. 22.1). Ordinal regression showed no significant dose effect using BCCT.core-based scores (*p* = 0.3). However, Harvard-based Maturation Effect Scores demonstrated greater deterioration following 3-week RT versus no RT (*p* = 0.036) and versus 5-day RT (*p* = 0.024).

## Discussion

This study provides the first comparative documentation of natural aesthetic trajectory in post-mastectomy breast reconstructions with and without autologous fat grafting. Three principal novel findings emerged: (1) non-grafted reconstructions demonstrated significant aesthetic deterioration between 1 and 6–12 months post-surgery (46% deteriorated, 4% achieved complete complaint resolution), occurring uniformly across reconstruction modalities; (2) fat grafting fundamentally reversed this trajectory, converting progressive decline into sustained improvement (88.1% complete complaint resolution) with large effect sizes (rank-biserial correlations −0.872 to −1.000 vs. +0.5 to +0.7 in controls); and (3) conventional 3-week radiotherapy produced greater aesthetic deterioration in non-grafted reconstructions versus 5-day ultra-hypofractionated regimens (*p* = 0.036 and *p* = 0.024), while providing greater potential for measurable improvement following fat grafting.

These findings establish that post-reconstructive aesthetic deterioration should be anticipated rather than considered aberrant, and that fat grafting functions as a regenerative intervention modifying biological maturation trajectory rather than simply providing volumetric correction. Universal deterioration across implant-based and autologous reconstructions (Kruskal-Wallis: BCCT.core *p* = 0.315; Harvard *p* = 0.156) suggests common pathophysiologic processes of scar contracture, tissue settling, and progressive fibrosis^19^, ^20^, ^21^ that fat grafting counteracts through adipose-derived stem cell-mediated regeneration.^12^, ^13^, ^14^, ^15^, ^16^, ^17^

Inclusion of a non-fat-grafted comparison cohort distinguishes this study from existing literature, where fat grafting outcomes are typically reported without comparative assessment.^15^, ^16^, ^17^ The marked divergence in complaint resolution rates (88.1% complete vs. 4%) underscores the clinical magnitude of fat grafting's trajectory-modifying effect.

Fat grafting produced consistent improvement across all reconstruction types and radiotherapy exposures, confirming broad therapeutic applicability. Neither reconstruction technique nor grafting session number influenced improvement magnitude. Irradiated reconstructions, particularly 3-week PMRT regimens, demonstrated greatest objective gains on BCCT.core analysis, likely reflecting poorer baseline aesthetics and greater capacity for measurable recovery. Harvard Scale assessments confirmed substantial improvement independent of radiotherapy status. Radiotherapy exposure did not affect grafting session number required, indicating irradiated tissues respond favorably to standard grafting volumes and techniques. BCCT.core analysis identified greater objective gains in irradiated breasts, consistent with inferior baseline appearance and fat grafting's ability to ameliorate radiation-induced textural and volumetric deficits.

Concordant subjective and objective improvements underscore fat grafting's multifactorial role in reconstructive optimization. Beyond volumetric augmentation, grafted adipose tissue, rich in adipose-derived stem cells and paracrine mediators—promotes microvascular regeneration, collagen remodeling, and fibrosis reduction.^12^ Greater objective improvement in irradiated breasts, including implant-based reconstructions, aligns with Pagliara et al. (2022), who reported histological normalization of irradiated capsules after repeated fat grafting, including reduced capsule thickness, decreased α-SMA expression, and enhanced ER-β positivity.^13^ Serial fat grafting partially reverses radiation-induced fibrosis and improves implant tolerance.^14^^,^^15^ These mechanisms are supported by measurable aesthetic improvement using both assessment methods.

### Natural deterioration in non-fat grafted reconstructions versus improvement observed in fat-grafted reconstructions

Cohort 2 demonstrated significant aesthetic deterioration between 1 and 6–12 months post-reconstruction (BCCT.core: *p* = 0.027, *r* = 0.5; Harvard: *p* < 0.001, *r* = 0.7). Non-fat-grafted cases showed 46% deterioration, 14% improvement, 4% complete resolution. This decline, likely reflecting scar contracture, tissue settling, implant/flap changes, or capsular contracture,^19^^––^^21^ occurred universally across modalities (Kruskal-Wallis: BCCT.core *p* = 0.315; Harvard *p* = 0.156). Conventional 3-week radiotherapy showed greater Harvard deterioration than no RT (*p* = 0.036) or 5-day RT (*p* = 0.024), consistent with radiation-induced fibrosis and atrophy.^12^^,^^14^

Conversely, Cohort 1 demonstrated active aesthetic improvement following fat grafting (r=−0.872 to −1.000, *p* = 0.006). This divergence (Mann-Whitney *p* < 0.001; effect sizes 0.41–0.84) indicates fat grafting converts progressive decline into sustained enhancement.^15^, ^16^, ^17^ Higher complaint resolution with fat grafting (88.1% complete, 11.9% partial) versus spontaneous resolution (4% complete, 10% partial) supports proactive, staged grafting in reconstruction algorithms, particularly in high-risk irradiated settings.^12^^,^^14^^,^^16^^,^^18^, ^19^, ^20^ These findings indicate "modest" non-grafted results may reflect successful limitation of decline rather than stability. Fat grafting acts as regenerative intervention modifying biological and aesthetic maturation of reconstructed breasts.^12^^,^^13^

### Reconstruction type and grafting frequency

Absence of significant differences in aesthetic outcomes between reconstruction types in both cohorts suggests fat grafting yields comparable outcomes regardless of modality and that natural scar maturation is universal. This aligns with De Blacam et al. and Kanchwala et al., who demonstrated reliable improvements with fat grafting across reconstructive settings, and Kwan et al. regarding natural scar development. The broader improvement detection by Harvard Scale in Cohort 1 likely reflects its sensitivity to three-dimensional contour refinements, whereas BCCT.core relies primarily on two-dimensional symmetry and color metrics.

One to two sessions in Cohort 1 mirrors literature frequencies, reflecting staged optimization. In implant-based reconstructions, fat grafting enhances coverage, soft-tissue thickness, and smoothness. Patel et al. and Salgarello et al. reported periprosthetic grafting improves contour and reduces revisions, consistent with our findings that one or two sessions achieved contour correction and rippling camouflage.

For autologous DIEP and LD flaps, fat grafting provided incremental refinement rather than transformation. De Blacam et al. and Losken et al. reported comparable gains in contour balancing and upper-pole fullness. Consistent one-grade improvements across DIEP and LD reconstructions reinforce fat grafting's role as complementary refinement rather than substitute for primary techniques.

### Influence of radiotherapy

Objective BCCT.core analysis revealed greater improvement in irradiated fat-grafted reconstructions, particularly following 3-week RT, likely reflecting correction of pre-existing radiation sequelae rather than superior regenerative response, consistent with reported histological and clinical softening of fibrotic irradiated planes. Non-irradiated tissues demonstrated smaller measurable gains, likely attributable to more favorable baseline and limited ceiling for improvement. The absence of correlation between radiotherapy dose/fractionation and number of grafting sessions suggests that once radiogenic changes stabilize, fat grafting efficacy is independent of fractionation scheme.

In non–fat-grafted reconstructions, deterioration detected by BCCT.core did not correlate with RT exposure, whereas Harvard scale measurements demonstrated significantly greater deterioration in 3-week regimen reconstructions. Collectively, these findings confirm more prominent negative sequelae following 3-week regimen compared with 5-day regimen or no RT exposure, thereby providing greater potential for aesthetic improvement through fat grafting in appropriate candidates.

### Limitations and future steps

Several limitations warrant consideration. Retrospective design introduces selection bias, and bilateral reconstructions (*n* = 10 Cohort 1, *n* = 2 Cohort 2) may affect statistical independence, though sensitivity analyses confirmed robustness. The small 5-day ultra-hypofractionated radiotherapy subgroup (*n* = 3 Cohort 1, *n* = 9 Cohort 2) precludes definitive fractionation comparisons; findings should be considered exploratory.^12^^,^^14^

The study prioritized objective aesthetic assessment (BCCT.core, Harvard Scale) over patient-reported outcome measures.^6^^,^^9^^,^^10^ While PROMs such as BREAST-Q provide insight into patient satisfaction⁶,complaint resolution demonstrated substantial differences (88.1% complete Cohort 1 vs. 4% Cohort 2). Future studies integrating PROMs with objective assessments would provide comprehensive evaluation.

The 6–12 month follow-up does not capture long-term volumetric retention or late radiation effects. Two-dimensional BCCT.core photography may underestimate three-dimensional refinements, necessitating complementary Harvard Scale grading (κ=0.61) .^9^^,^^10^ Histologic validation of regenerative mechanisms remained beyond scope.^12^^,^^13^

Despite these limitations, Cohort 2 inclusion addresses a critical literature gap by documenting natural post-reconstructive aesthetic trajectory and establishing fat grafting's trajectory-modifying effects^15^, ^16^, ^17^ Future multicenter prospective studies with larger radiotherapy subgroups, extended follow-up, three-dimensional imaging, and integrated PROMs are warranted.

## Conclusion

Post-reconstructive aesthetic deterioration occurred in 46% of non-grafted reconstructions, challenging stability assumptions. Fat grafting reversed this trajectory, achieving 88.1% versus 4% complete complaint resolution, consistently across reconstruction modalities and radiotherapy exposures. Irradiated reconstructions, particularly 3-week fractionation, demonstrated greatest improvement. Fat grafting functions as regenerative intervention through adipose-derived stem cell-mediated regeneration, collagen remodeling, and fibrosis reduction, extending beyond volumetric correction. Documentation of natural deterioration patterns supports proactive staged incorporation into reconstructive algorithms, particularly for irradiated patients. Future studies integrating patient-reported outcomes, extended follow-up, and histologic validation will refine selection and optimize protocols.

## Disclosure of interest and data availability statement

No potential competing interest was reported by the authors.

No funding was received.

Data sharing is not applicable to this article.

## Ethical approval

This study was approved as a clinical audit by the Audit and Clinical Governance Committee at Addenbrooke's Hospital, Cambridge University Hospitals NHS Foundation Trust (Project ID7249, PRN 13,249). All procedures were performed in accordance with institutional ethical standards and the Declaration of Helsinki.
